# Development of a Zero-Stagnant-Water Purification System Based on Smart Series–Parallel Control of Dual RO Membranes

**DOI:** 10.3390/membranes16050155

**Published:** 2026-04-23

**Authors:** Mei Ma, Bin Huang, Lingling Mei, Kan Huang, Ke Xing, Lida Liao

**Affiliations:** 1 School of Electric and Information Engineering, Yangzhou Polytechnic Institute, Yangzhou 225002, China; maimei55768194@126.com (M.M.); mll1090685874@163.com (L.M.); 2 School of Electrical and Mechanical Engineering, Adelaide University, Adelaide, SA 5000, Australia; 3 School of Future Transportation, Guangzhou Maritime University/Guangzhou Transportation University (Preparatory), Guangzhou 510725, China; 4 School of Energy and Power Engineering, Changsha University of Science and Technology, Changsha 410004, China

**Keywords:** reverse osmosis, membrane water treatment, intermittent operation, process control, water stagnation, series–parallel configuration

## Abstract

Intermittently operated, tankless reverse osmosis (RO) systems are widely used in decentralized and point-of-use applications, yet water stagnation during idle periods remains a critical challenge, leading to degraded water quality, accelerated fouling, and performance loss. This study presents an experimentally validated engineering solution that eliminates stagnant water in intermittently operated RO systems. A dual-membrane RO configuration with flexible series–parallel switching was developed, enabling membranes to alternate between production and flushing modes. An adaptive control strategy, integrated into the system hardware, regulates membrane switching and flushing based on real-time feed-water quality. The proposed configuration and control framework was evaluated under representative intermittent operating conditions. Experimental results show that the zero-stagnant-water strategy effectively prevents residual water accumulation during shutdown and maintains stable permeate quality, with total dissolved solids consistently below 10 mg/L. Long-term testing further demonstrates reduced membrane fouling and slower performance degradation compared with conventional fixed-operation schemes, resulting in enhanced desalination efficiency and operational stability. Owing to its modular design and simple control logic, the proposed approach is readily transferable to decentralized and point-of-use membrane water treatment systems requiring reliable, high-quality water under intermittent operation.

## 1. Introduction

Reverse osmosis (RO) is one of the most important membrane-based water purification technologies and is widely deployed for desalination, industrial wastewater reuse, and household drinking water treatment. Its effectiveness arises from the selective permeability of thin-film composite membranes, which can efficiently remove dissolved salts, organic contaminants, and microorganisms. The rapid global rise in water demand has further driven research toward improving the reliability and sustainability of RO systems [1,2,3]. A standard RO unit generally involves multiple pretreatment steps, a high-pressure pump, a semi-permeable membrane module, and post-treatment processes, which together reduce the total dissolved solids (TDS) to potable levels [4,5].

Despite its efficiency advantages, the performance of RO systems is hindered by persistent operational issues. Fouling by natural organic matter, colloids, and microbial communities significantly decreases water permeability and increases energy requirements [6,7,8,9,10]. The gradual accumulation of biofilms and inorganic deposits accelerates membrane degradation, raises cleaning frequency, and can result in the deterioration of permeate quality. These challenges are particularly critical in point-of-use purifiers, where intermittent operation and stagnant water zones may trigger short-term degradation in water quality.

In addition to operational challenges such as fouling and stagnation, the intrinsic chemical stability of the selective layer in thin-film composite (TFC) membranes plays a crucial role in determining long-term performance. Conventional polyamide-based TFC membranes are known to suffer from chemical degradation under extreme pH conditions, which limits their durability and operational lifespan. Recent studies have demonstrated that alternative selective-layer chemistries, particularly polyurea-based membranes, exhibit significantly enhanced resistance to both acidic and alkaline environments. For example, chemically robust hollow fiber TFC membranes incorporating polyurea selective layers have been shown to maintain high rejection performance even after prolonged exposure to harsh pH conditions, highlighting their potential for improving membrane longevity and reliability [11]. These advances indicate that material innovation, when combined with intelligent process control strategies, provides a synergistic pathway toward enhancing the stability and sustainability of RO systems, which is further explored in this study through process-level control strategies.

In recent years, progress in sensing technologies, Internet of Things (IoT) platforms, and artificial intelligence (AI) has enabled the development of intelligent, data-driven RO systems. Integrated sensors allow for the continuous monitoring of pressure, conductivity, turbidity, and flow, facilitating the early detection of fouling, membrane wear, and feedwater fluctuations [12,13,14,15,16,17]. AI-assisted control algorithms further support adaptive operation, enabling the optimization of cleaning cycles and reduction in unnecessary water waste. Beyond operational control, researchers have emphasized the importance of membrane lifecycle management. Strategies such as membrane recycling, selective refurbishment, and environmentally conscious disposal are being actively explored to reduce waste generation and reinforce sustainability goals within the desalination sector [18,19,20,21,22,23].

The objective of this study was to develop and experimentally validate a dual-membrane RO system with intelligent series–parallel switching to eliminate stagnant water and enhance long-term operational stability. The key research questions addressed include: (i) how dynamic membrane configuration influences water quality during intermittent operation, (ii) how adaptive flushing strategies mitigate fouling, and (iii) whether the proposed system can sustainably maintain high rejection performance under realistic household conditions.

## 2. Literature Review

Recent RO research can be organized into four major themes. These themes relate to (1) mechanisms of fouling and associated mitigation strategies, (2) intelligent monitoring and sensor-assisted control, (3) IoT-enabled predictive maintenance, and (4) membrane lifecycle management and sustainability.

### 2.1. Fouling Mechanisms and Cleaning Strategies

Fouling remains a fundamental challenge that limits the long-term performance of RO systems. Accumulated organic matter, particulate materials, and microbial biofilms disrupt mass transfer and reduce permeate flux. Even with optimized pretreatment, fouling is consistently reported as a primary cause of performance decline in desalination and potable water purification [8,9]. Early investigations by Flemming revealed that biofilms exhibit strong structural resilience, making them difficult to eliminate using standard physical or chemical methods [10].

To address fouling, chemical cleaning strategies have become an essential component of RO system management. Li and Elimelech demonstrated that the effectiveness of chemical cleaning is highly dependent on the nature of foulants and the compatibility of cleaning reagents with specific membrane materials [12]. More recent work has highlighted the potential of controlled chlorination to inhibit microbial growth while maintaining membrane integrity when appropriately dosed [23]. These findings emphasize the importance of tailoring cleaning protocols to site-specific fouling characteristics.

### 2.2. Intelligent Monitoring and Sensor-Assisted Control

Advances in sensing and automation technology have significantly improved RO operational stability. Measurements of transmembrane pressure, conductivity, turbidity, and flow rate serve as real-time indicators of membrane condition and feedwater variability. Intelligent control systems that incorporate these measurements are capable of adjusting operational parameters automatically and detecting performance deviations at an early stage [13,14,15].

AI-based predictive models have further expanded the potential of adaptive RO control. Machine learning algorithms have been applied to predict fouling progression, optimize cleaning cycles, and enhance control accuracy in both water purification and wastewater treatment contexts [4,5,18]. These models improve energy efficiency and reduce water waste while maintaining output quality. The integration of AI has therefore become a promising direction for next-generation household and industrial RO units.

### 2.3. IoT-Enabled Predictive Maintenance

IoT platforms support continuous system monitoring and remote supervision, enabling the long-term observation of water quality and operational conditions [14,15,16]. Compared with conventional maintenance practices that rely on fixed intervals, predictive maintenance utilizes data-driven diagnostics to determine cleaning requirements based on performance trends. This approach provides more precise scheduling, reduces unnecessary cleaning, and mitigates membrane stress caused by excessive cycling.

Prototype systems equipped with IoT-based sensors and automated control valves have demonstrated strong potential for real-time diagnostic capabilities. Such systems can identify the onset of fouling, adjust operating conditions, and maintain permeate quality with minimal user input [17]. The scalability of IoT technologies suggests broad applicability in both domestic and industrial RO settings.

### 2.4. Sustainability and Membrane Lifecycle Management

With the expansion of RO deployment worldwide, membrane waste has emerged as an important environmental concern. Recent studies highlight the potential of circular-economy strategies that include membrane recycling, repurposing for low-pressure filtration, and systematic lifecycle management [1,22]. These practices reduce waste volume and provide economic benefits through the extension of membrane utility.

Improvements in cleaning strategies and operational control also contribute to sustainability. Optimized chemical-cleaning procedures, improved cleaning-cycle management, and intelligent process control help lower chemical discharge, minimize brine waste, and reduce energy consumption [19,20]. Comparative environmental assessments indicate that long-term sustainability requires balancing energy use, operational cost, and environmental impact in desalination and water-reuse applications [2,3].

## 3. System Development and Framework Description

### 3.1. System Architecture and Configuration

**Figure 1:**
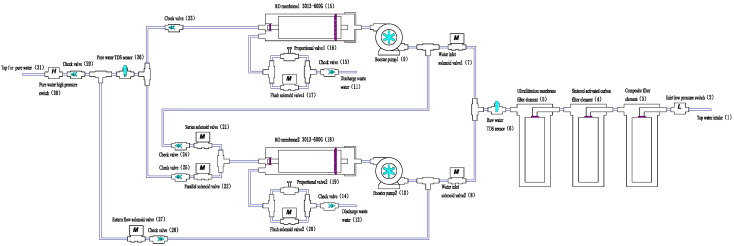
Framework of the S_PDRO system.

The overall configuration of the proposed series–parallel intelligent switching dual-RO membrane zero-stagnant-water (S_PDRO) water purification system is illustrated in Figure 1. The system integrates pre-filtration, pressurization, reverse osmosis, and intelligent control modules to ensure efficient purification and stable water production. Its main functional components include:

- Three-stage pre-filtration: As RO membranes are sensitive to free chlorine ions that can degrade performance and shorten lifespan, pre-filtration is essential. The first two stages employ a composite PP cotton and activated carbon filter (3) and a sintered activated carbon filter (4) to remove free chlorine and organic matter. The third stage uses an ultrafiltration membrane (5) to eliminate suspended solids, bacteria, viruses, and proteins, thereby protecting the RO membranes and improving feedwater quality.
- Booster pumps (9, 10): The RO process requires sufficient hydraulic pressure to drive water molecules through the semi-permeable membrane. The booster pumps provide the necessary pressure to maintain stable operation and consistent flow under varying inlet conditions.
- Reverse osmosis membranes (15, 18): Only water molecules can permeate the RO membranes, effectively producing high-purity water. Two 600G RO membranes are configured in a series–parallel hybrid arrangement, enabling high throughput, direct dispensing, and a strong, continuous flow. The RO membranes used in this study were commercially available elements manufactured by Vontron. The membrane material is a polyamide thin-film composite (TFC). Under standard test conditions (pressure: 100 psi; temperature: 25 °C; feed: 250 ppm NaCl; pH: 6.5–8.5; recovery: 50%), each element has a nominal permeate capacity of 600 GPD (2.27 m^3^/day) and a salt rejection rate of 98% (minimum 96%).
- Solenoid valves: Seven solenoid valves, controlled by the main control board, regulate water flow and enable automatic switching between series and parallel operating modes.
- Sensors and control: The system includes high- and low-pressure switches and a total dissolved solids (TDS) sensor. TDS measurements were obtained using the built-in system sensor and cross-validated with a handheld TDS meter (Xiaomi, model XMTDS01YM, Beijing, China), with an accuracy of ±5%. Pre-filtration cartridges include an ultrafiltration cartridge (SER-011UF, Suer, Shanghai, China), activated carbon cartridge (F-CTO-10, Songquan, Taiwan, China), and composite filter cartridge (FPC-10-S, Songquan, Taiwan, China). These sensors provide feedback to the control board for real-time monitoring and adaptive regulation of pressure, water quality, and system operation.

### 3.2. Operation Logic and Workflow

The system operates under an intelligent control framework designed to optimize both water quality and operational efficiency. Upon startup, it first runs in series mode to flush residual water from the RO membrane housings, achieving a zero-stagnant-water condition. After approximately 30 s, it automatically switches to parallel mode to increase water production capacity and maintain a strong, continuous output. The selection of the 30 s switching interval is based on experimental observation rather than arbitrary choice. After prolonged stagnation (8 h), the TDS within the membrane housing reaches a peak value. Upon restart, the permeate TDS decreases rapidly and stabilizes within approximately 25 s. Therefore, a 30 s duration was selected to ensure complete displacement of stagnant water and sufficient flushing of the membrane housing. This duration is consistent with the hydraulic residence time of the system. When powered off, the system reverts to series mode within 30 s, allowing backflow to replace the water in the membrane housings and prevent stagnation.

An adaptive flushing strategy is implemented based on the total dissolved solids (TDS) level of the feed water. Poorer inlet water quality triggers higher flushing frequency and longer flushing duration, thereby protecting the RO membranes and extending their service life. Screen purified water TDS is continuously monitored by sensors, while an LCD screen provides real-time water quality data and filter replacement reminders. The system operates through four principal states, as illustrated below.

State 1 Within the first 30 s after start-up

**Figure 2:**
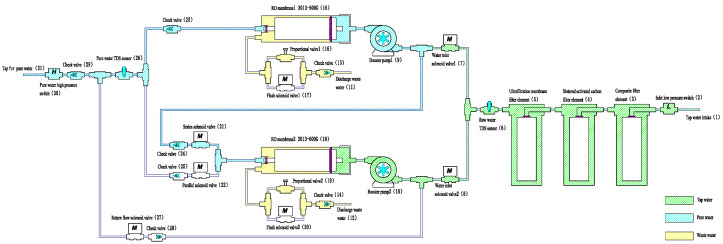
RO membranes 1 and 2 in series during the first 30 s after start-up.

As shown in Figure 2, during the initial 30 s, the two RO membranes operate in series to eliminate residual water within the membrane housings. When the purified water high-pressure switch turns off (indicating the faucet is on), the inlet solenoid valve and booster pump are activated. Feed water passes through the pre-filters and sequentially through RO membrane 1 and RO membrane 2. This configuration allows RO membrane 1 to further purify the residual water in RO membrane 2, ensuring low-TDS output and achieving the zero-stagnant-water function.

State 2 After 30 s from start-up

**Figure 3:**
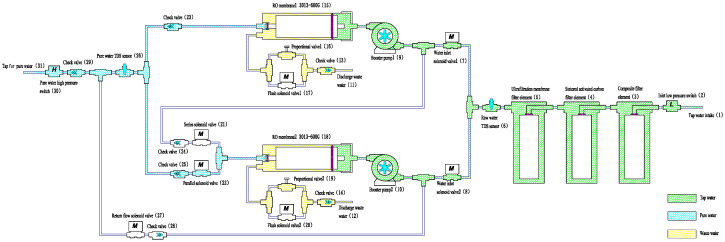
Parallel operation of the two RO membranes after 30 s of startup.

After 30 s, the system switches from series to parallel operation to enhance water production. As demonstrated in Figure 3, the parallel solenoid valve opens, and both RO membranes simultaneously supply purified water to the faucet. The combined output significantly improves the flow rate, suitable for high-demand usage scenarios.

A mass balance analysis was conducted to clarify system operation. In State 1 (series mode, without flushing), one unit of feed water produces approximately 0.75 units of permeate. In State 2 (parallel mode with flushing), two units of feed water produce approximately 1.5 units of permeate. The remaining fraction is discharged as concentrate, consistent with a permeate-to-wastewater ratio of approximately 3:1. This ratio is further supported by experimental results presented in the following section, demonstrating stable long-term performance under this operating condition.

State 3 Within 30 s after shutdown

**Figure 4:**
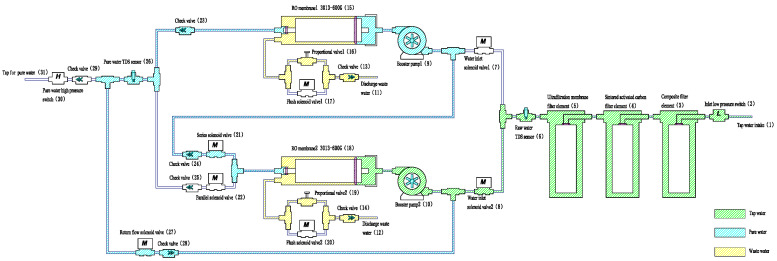
Return to series operation of the RO membranes 30 s after shutdown.

When the system detects the cessation of water use, the control logic closes selected solenoid valves and activates the return and series flow paths (see Figure 4). Purified water from RO membrane 2 flows back into the housing of RO membrane 1, displacing residual feed and wastewater, reducing osmotic pressure differences, and preventing stagnant water formation. During post-production recirculation, the membrane module is filled predominantly with low salinity permeate water. This reduces the concentration gradient across the membrane, thereby mitigating concentration polarization and osmotic back-diffusion during idle periods. The system then shuts down and enters standby mode. If water resumes within 30 s, it directly returns to parallel operation.

State 4 Flushing

**Figure 5:**
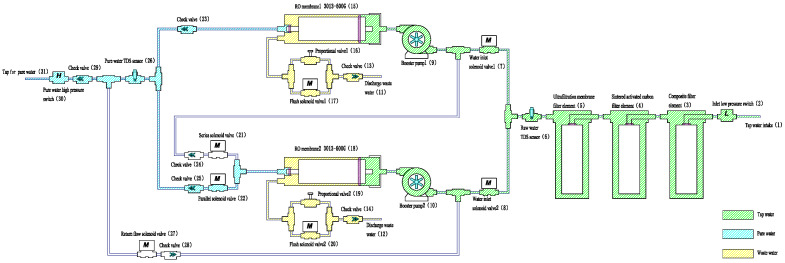
Initiation of flushing strategy during parallel RO operation.

During parallel operation, the system periodically performs a flushing process by adjusting the wastewater and flushing solenoid valves to temporarily increase wastewater flow and remove surface deposits from the RO membranes (as illustrated in Figure 5). The flushing frequency and duration are determined by the feedwater TDS level, as summarized in Table 1. Even with a wastewater ratio of 3:1, this adaptive flushing strategy effectively prolongs membrane lifespan while maintaining purification efficiency.

In summary, the coordinated operation of the four states, together with the adaptive flushing mechanism, ensures high-efficiency purification, extended RO membrane longevity, and consistent delivery of high-quality water under varying feedwater conditions.

### 3.3. Control Board Hardware Design

The proposed water purification system adopts a three-stage pre-treatment configuration. Raw water sequentially passes through a composite filter cartridge, a sintered activated carbon filter, and an ultrafiltration membrane to remove suspended solids and organic contaminants prior to reverse osmosis (RO) treatment. The system integrates two pressure pumps and two RO membranes. Through coordinated control of multiple solenoid valves, the RO membranes can operate in either series or parallel modes, allowing flexible adaptation to different operating conditions.

During system start-up, the RO membranes operate in series for the first 30 s to eliminate stagnant water within the membrane housings. The system then automatically switches to parallel operation to increase the permeate flow rate and satisfy high water demand. Upon shutdown, a series recirculation mode is reactivated for 30 s, flushing residual water from the membrane housings with purified water. This operational strategy effectively prevents stagnant water formation and ensures consistent permeate quality.

Water quality data acquired from the raw water total dissolved solids (TDS) sensor is used to dynamically regulate the wastewater valve and implement adaptive backwashing strategies. Under a preset wastewater ratio of 3:1 (permeate to concentrate), the system achieves a balance between water recovery and membrane fouling control. The reported 3:1 permeate-to-concentrate ratio is supported by mass balance analysis and experimental validation illustrated in the following section, demonstrating stable operation without rapid scaling. This approach reduces water consumption while facilitating the removal of accumulated contaminants on the RO membrane surface, thereby extending the membrane service life. A downstream TDS sensor continuously monitors permeate quality, and the measured values are displayed in real-time on an LCD interface. When abnormal water quality is detected, the system issues alert to prompt timely filter replacement and prevent the consumption of non-compliant water.

**Figure 6:**
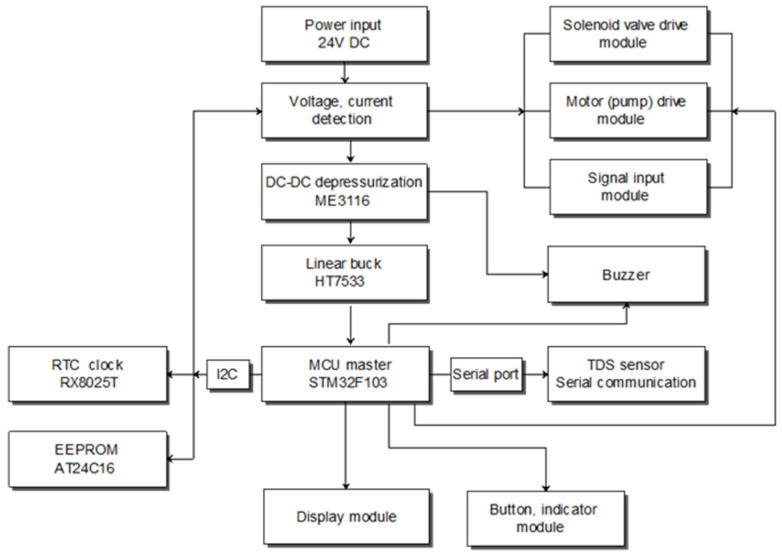
Framework of the hardware system.

The central control unit consists of a power management module, a microcontroller unit (MCU), and dedicated drive circuits for solenoid valves and motors (as shown in Figure 6). The power management module provides voltage regulation and electrical monitoring, while the MCU serves as the core controller. It acquires TDS data via serial communication and time information via the I^2^C interface and subsequently controls valve switching and pump operation. These functions enable zero-stagnant-water operation and RO membrane backwashing while maintaining water quality and improving overall water-use efficiency. System status, alarms, and operating conditions are communicated to the user through an LCD, indicator LEDs, and an audible buzzer. The detailed circuit diagrams of each module are provided in Appendix A.

- Power Supply and Electrical Monitoring

The system operates with a 24 V power input. Protective components, including a fuse, a Schottky diode, and a transient voltage suppression (TVS) diode, are incorporated to prevent damage from reverse polarity and voltage transients. After capacitive filtering, the stabilized input voltage supplies the downstream circuitry. An INA226 monitoring chip continuously measures system voltage and current, enabling the real-time assessment of electrical load conditions. Overcurrent and overvoltage protection mechanisms are triggered when abnormal conditions are detected to ensure safe operation.

The 24 V input is first stepped down to 6 V using an ME3116 DC–DC buck converter to supply the motor drive circuits and buzzer. A subsequent HT7533 linear regulator reduces the voltage to 3.3 V for the MCU and low-power electronic components. This two-stage regulation scheme enhances power stability and minimizes electrical noise, thereby improving the reliability of sensitive control and sensing circuits.

- Main Control Unit

An STM32F103C8T6 microcontroller is employed as the main control unit due to its adequate computational capability, rich peripheral resources, and suitability for real-time control applications. An external crystal oscillator provides a stable clock source, ensuring reliable operation of serial communication interfaces and the I^2^C bus.

User interaction is facilitated through a set of push buttons and indicator LEDs. The buttons allow manual operations such as mode selection and parameter adjustment, while the LEDs provide intuitive visual feedback on system states, including water production, standby, flushing, and alarm conditions. TDS sensors communicate with the MCU via TTL-level serial interfaces to ensure stable and efficient data transmission. Additionally, reserved serial communication ports are provided to support future system expansion and peripheral integration.

- Display, Real-Time Clock, and Data Storage

The display and data management subsystem includes an RX8025T real-time clock (RTC), an AT24C16 EEPROM, an LCD module, and a buzzer. RTC provides accurate timekeeping and supports low-power sleep modes to reduce overall energy consumption. The EEPROM stores operational data such as cumulative water usage, filter service duration, and system logs, ensuring data retention during power interruptions.

The LCD communicates with the MCU via an SPI interface and presents real-time information, including operating status, water quality indicators, and remaining filter lifespan. A passive buzzer powered by the 6 V supply generates audible alarms in response to abnormal water quality, filter replacement requirements, or system faults, enabling timely user intervention.

- Input and Output Interfaces

The input interface consists of three identical signal-conditioning circuits, each based on a transistor, which converts 24 V sensor signals to 3.3 V logic-level signals compatible with the MCU. High-pressure and low-pressure switches are connected to these inputs to provide real-time feedback on system operating conditions. The MCU interprets these signals to determine system status and execute appropriate control actions.

The output interface comprises six identical driver circuits using WSD4050 NMOS transistors to control solenoid valves. When a high-level control signal is applied by the MCU, the NMOS transistor conducts and actuates the corresponding solenoid valve, enabling precise control of flow paths under different operating modes.

- Motor Driver Module

The motor drive module consists of two identical circuits, each incorporating a WSD4050 NMOS transistor and a totem-pole driver configuration. Pulse-width modulation (PWM) is employed to regulate the power supplied to the booster pumps, allowing for accurate control of pump speed. This enables the dynamic adjustment of water pressure and flow rate in response to water production demand and membrane flushing requirements, thereby improving energy efficiency and operational stability.

- TDS Sensor Circuit Design

The TDS sensing subsystem integrates two temperature-compensated TDS sensors, enabling independent measurement of raw water and permeate TDS values. NTC-based temperature compensation ensures measurement accuracy under varying thermal conditions. A linear voltage regulator stabilizes the supply voltage at 3.3 V, providing a low-noise power source for the sensors and associated circuitry.

An HT66F019 microcontroller, operating with manufacturer-provided firmware, is used for sensor control and signal processing, ensuring stable measurement performance and reliable data handling. TDS data are transmitted to the main MCU via serial communication in real-time, providing a robust basis for water quality evaluation and adaptive control of flushing and operating strategies.

### 3.4. Logic Control and Timing Relationships

Building upon the hardware architecture and circuit design described in the preceding section, the following part focuses on the software control strategy of the water purification system. The embedded control program coordinates sensing, decision-making, and actuation across multiple functional modules, ensuring stable operation, water quality safety, and efficient membrane utilization. By integrating real-time sensor feedback with time-based state transitions, the software enables adaptive operation under varying water demand and feedwater quality conditions.

After system initialization, the microcontroller continuously acquires raw-water and permeates TDS data via serial communication while concurrently executing the display update, key input detection, and buzzer alarm routines. In parallel, the control logic monitors supply voltage, operating current, and inlet pressure through dedicated sensors to verify that the electrical and hydraulic conditions remain within predefined safety thresholds. When abnormal voltage, current, or pressure conditions are detected, the controller immediately disables all actuators and triggers an alarm to protect the system.

Once the monitored electrical and hydraulic parameters satisfy the operating requirements and a water demand is detected (i.e., the outlet is opened), the controller initiates water production and starts accumulating the operating time. During the initial startup phase, both reverse osmosis (RO) membranes operate in a series configuration for 30 s. This stage stabilizes the hydraulic conditions and eliminates stagnant water inside the membrane housings. After this period, the system automatically switches to a parallel operating mode by actuating the corresponding solenoid valves and booster pumps, thereby increasing the permeate flow rate while maintaining continuous TDS monitoring of both feedwater and purified water.

To mitigate membrane fouling and extend membrane service life, an adaptive flushing strategy is implemented during parallel operation. The flushing interval and duration are dynamically adjusted according to the measured feedwater TDS level, with higher TDS values triggering more frequent and longer flushing cycles. This strategy balances water recovery and membrane protection without interrupting normal water production.

When water demand ceases, the controller resets the production timer and transitions the system into a post-production recirculation stage. The RO membranes are again operated in series for approximately 30 s, during which purified water is used to displace residual water in the membrane housings, thereby ensuring a “zero-stagnant-water” condition. After completion of this recirculation process, all pumps and solenoid valves are deactivated, operational data are stored in non-volatile memory, and the system enters standby mode.

The overall control logic and timing relationships are summarized in the following pseudocode. A detailed description of the logic control flow is presented in Appendix B.

*//Main control loop (simplified pseudocode)*

*Initialize system modules and timers;*

*while (system is powered) {*

*Read raw-water TDS and permeate TDS;*

*Monitor voltage, current, and pressure sensors;*

*Update display and process key inputs;*

*if (electrical or pressure fault detected) {*

*Disable all pumps and solenoid valves;*

*Trigger alarm;*

*Enter standby mode;*

*continue;*

*}*

*if (water demand detected) {*

*standbyTimer = 0;*

*produceTimer += Δt;*

*if (produceTimer < 30 s) {*

*//Startup: series operation*

*Enable pumps;*

*Open series valves;*

*Close parallel and recirculation valves;*

*} else {*

*//Normal production: parallel operation*

*Enable pumps;*

*Open parallel valves;*

*Close series and recirculation valves;*

*Execute adaptive flushing based on raw-water TDS;*

*}*

*} else {*

*produceTimer = 0;*

*standbyTimer += Δt;*

*if (standbyTimer < 30 s) {*

*//Post-production recirculation*

*Enable pumps;*

*Open series and recirculation valves;*

*Close parallel valves;*

*} else {*

*//Standby mode*

*Disable all pumps and solenoid valves;*

*Store operational data;*

*}*

*}*

*Delay(Δt);*

*}*

## 4. Experimental Study and Discussion

**Figure 7:**
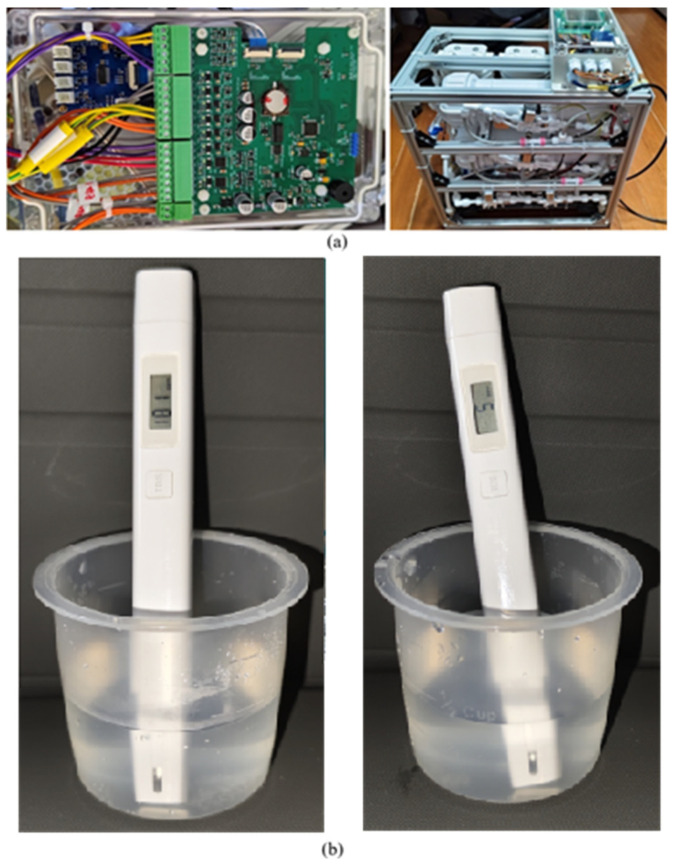
Prototype (**a**) and measurement kit (**b**).

The control circuit board was integrated into the RO purification system, connecting all sensors, solenoid valves, and motors. A 24 V, 6.5 A switching power supply was used for system operation. As illustrated in Figure 7a, the testing platform was constructed using 2020 aluminum profiles, providing high rigidity, modularity, and ease of assembly. The purified water quality was evaluated using a handheld TDS meter, as shown in Figure 7b. The inlet TDS value was 187 mg/L, and the permeate TDS was 4 mg/L, confirming the excellent purification performance of the developed system. The feedwater salinity (~200 mg/L) was selected to represent typical household drinking water conditions. Future work will extend testing to higher salinity levels (e.g., brackish water) to evaluate system robustness under more demanding conditions.

To verify the effectiveness of the zero-stagnant-water design, comparative experiments were conducted under two conditions: (1) with the zero-stagnant-water function disabled (control group) and (2) with it enabled (experimental group). For both tests, one sample of purified water was collected hourly, and its TDS value was measured. The experiments were performed at an indoor temperature of 19–25 °C, with a feedwater TDS of 189 mg/L and a water temperature of 20 °C.

**Figure 8:**
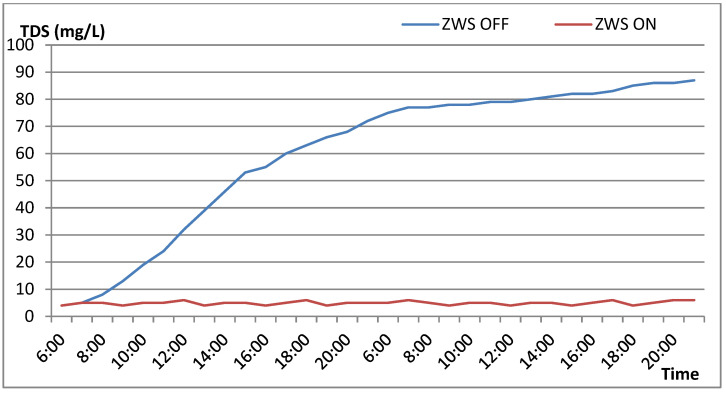
Time series of permeate TDS with ZSW ON/OFF.

As plotted in Figure 8, the TDS of the control group increased rapidly within the first few hours, rising above 60 mg/L after several hours and approaching its maximum after overnight stagnation. In contrast, when the zero-stagnant-water function was enabled, the TDS remained consistently below 10 mg/L throughout the entire test period. These results clearly demonstrate the effectiveness of the proposed design in eliminating stagnant water and maintaining consistently high-quality purified water.

To assess the long-term performance of the intelligent flushing strategy, two identical purification units—designated as Purifier 1 and Purifier 2—were tested under identical operating conditions. Purifier 1 implemented the intelligent flushing algorithm, while Purifier 2 served as a control without it. Both operated at a wastewater-to-pure-water ratio of 3:1. The membrane performance was evaluated based on the salt rejection rate, calculated as:


$$Desalination\;ratio=\frac{\left({TDS}_{feed}-{TDS}_{permeate}\right)}{{TDS}_{feed}}\times 100\%$$

For example, with a feedwater TDS of 187 mg/L and a permeate TDS of 4 mg/L, the initial rejection rate was 97.86%. To accelerate membrane aging, each purifier was operated with a daily water production of 100 L, approximately ten times that of a typical household. The rejection rate of both systems was recorded weekly over a 24-week test period.

**Figure 9:**
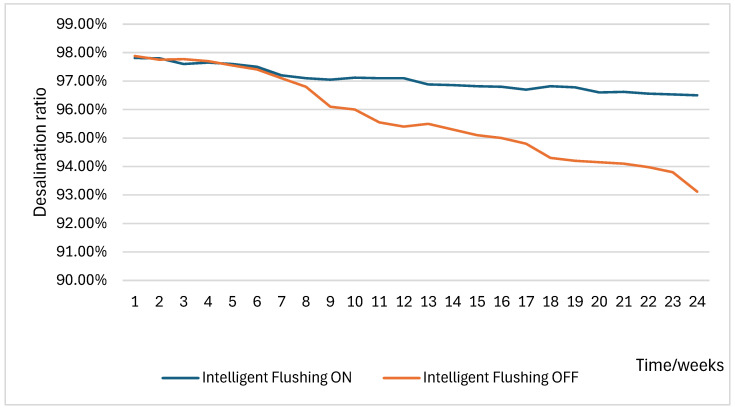
Desalination ratios with and without intelligent flushing.

The results, shown in Figure 9, indicate that the RO membrane equipped with the intelligent flushing strategy maintained a higher rejection rate and exhibited a slower rate of decline compared to the control. After 24 weeks, the membrane in the intelligent flushing system retained a rejection rate of 96.5%, whereas the control dropped to 93.1%. These findings confirm that the intelligent flushing strategy significantly extends membrane lifespan and enhances purification stability.

All experimental results demonstrate that the proposed system achieved its intended design objectives. The purifier exhibited stable performance, efficient operation, and user-friendly functionality, supporting its feasibility for large-scale application and commercialization.

## 5. Concluding Remarks

This study presents the development and evaluation of a microcontroller-based intelligent reverse osmosis (RO) water purification system that integrates advanced control strategies to enhance performance, reliability, and water-use efficiency. The system was designed to address key issues in conventional household and small-scale purification devices, particularly those related to stagnant water contamination, excessive flushing waste, and shortened membrane lifespan.

Through the implementation of a dynamic dual-RO configuration, the system achieves genuine zero-stagnant-water operation. By intelligently switching between serial and parallel membrane connections during different phases of water production, the system ensures that the initial effluent already meets the standard for purified water. Furthermore, the design enables the reuse of flushing water from the first RO membrane, significantly reducing the overall water wastage and improving resource utilization efficiency.

An adaptive flushing algorithm was also developed to maintain the long-term stability of the RO membranes. By continuously monitoring the feedwater TDS, the system automatically adjusts both the frequency and duration of the flushing process based on real-time water quality. This strategy effectively slows down membrane degradation, extends operational life, and sustains a high desalination rate throughout prolonged use. The balance achieved between membrane protection and water conservation underscores the system’s intelligent control capability.

Experimental results from integrated testing verified that the proposed system performed with high stability and excellent purification efficiency. The zero-stagnant-water function was proven to maintain consistently low TDS levels over extended periods, while the intelligent flushing mechanism preserved the RO membrane’s rejection rate over 24 weeks of continuous operation. Together, these results confirm that the system fulfills its design objectives and is feasible for large-scale practical deployment.

Overall, this work demonstrates a technically robust, economically viable, and environmentally sustainable approach to smart water purification. The proposed system not only enhances user experience by providing immediate access to high-quality water but also contributes to the broader goal of sustainable water management. The findings offer valuable insights into future research and industrial applications in intelligent RO systems, providing a foundation for the next generation of resource-efficient, automated water purification technologies. Although the present study demonstrated improved anti-fouling performance through operational strategies, detailed post-mortem characterization of the membranes (e.g., SEM or AFM analysis) was not conducted. Such analyses would provide deeper insights into fouling mechanisms and structural changes. This limitation will be addressed in future work.

## Figures and Tables

**Table 1:** Adaptive flushing strategy based on feedwater TDS level.

**Feedwater TDS (mg/L)**	**Flushing Duration**	**Flushing Interval (During Parallel Operation)**	**Flushing Frequency**
<50	None	—	No flushing
50–100	1 s	Every 30 s	moderate
100–150	2 s	Every 30 s	moderate–high
150–250	3 s	Every 30 s	high
250–500	5 s	Every 30 s	very high

**Figure A1:**
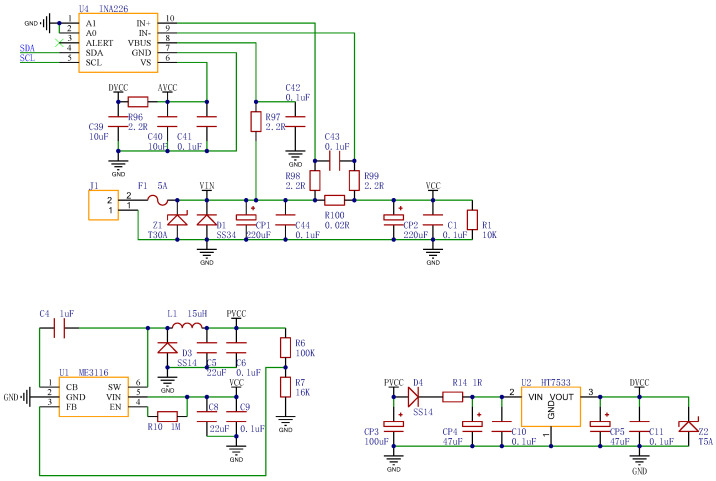
Power supply and electrical monitoring.

**Figure A2:**
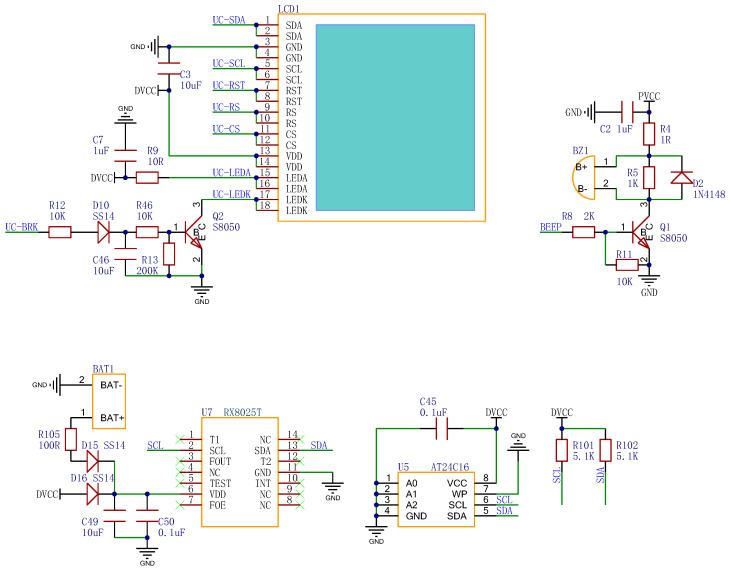
Display, real-time clock, and data storage.

**Figure A3:**
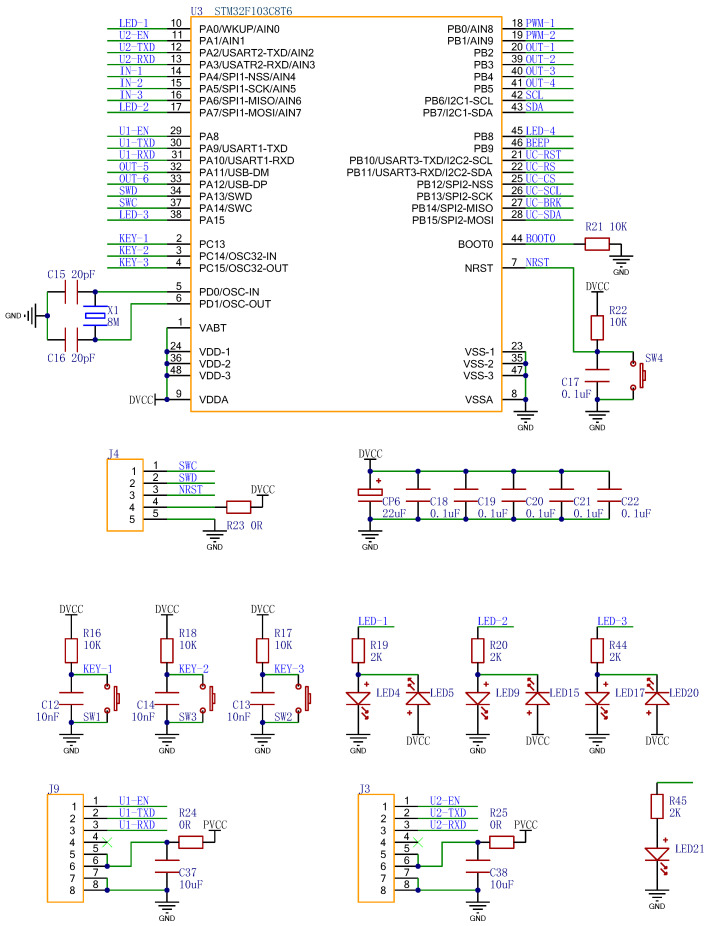
Main control unit.

**Figure A4:**
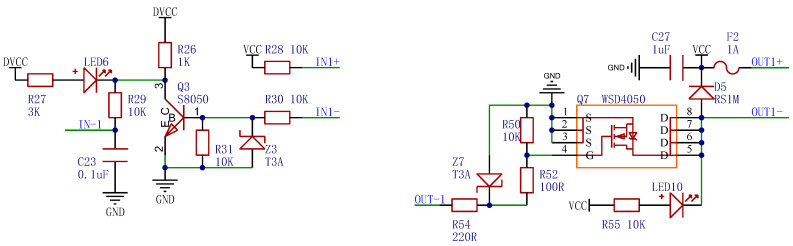
Input and output interfaces.

**Figure A5:**
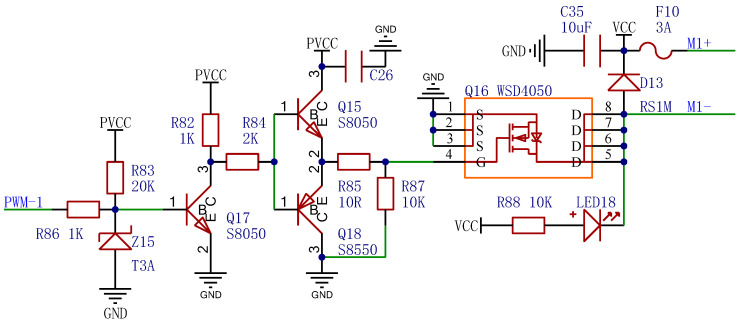
Motor driver module.

**Figure A6:**
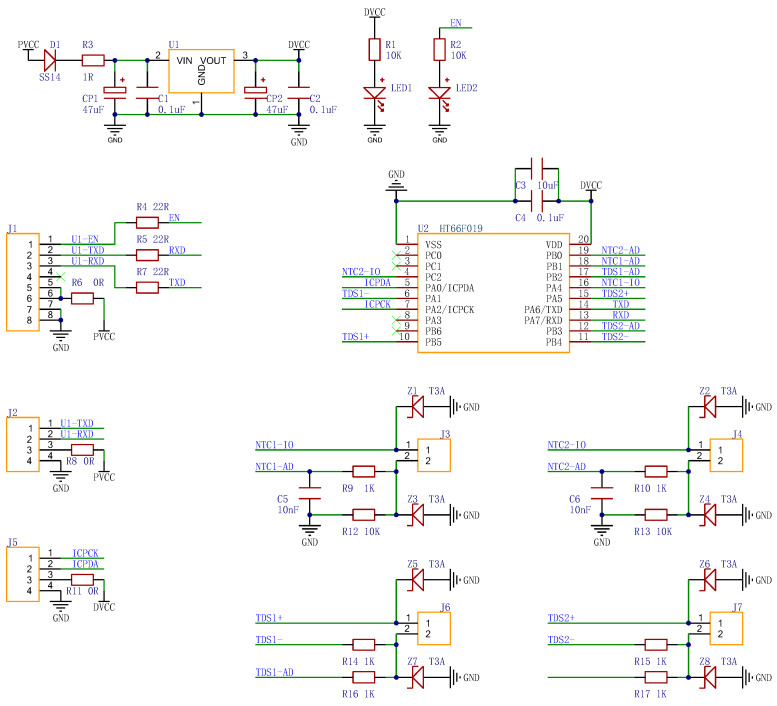
TDS sensor circuit design.

**Figure A7:**
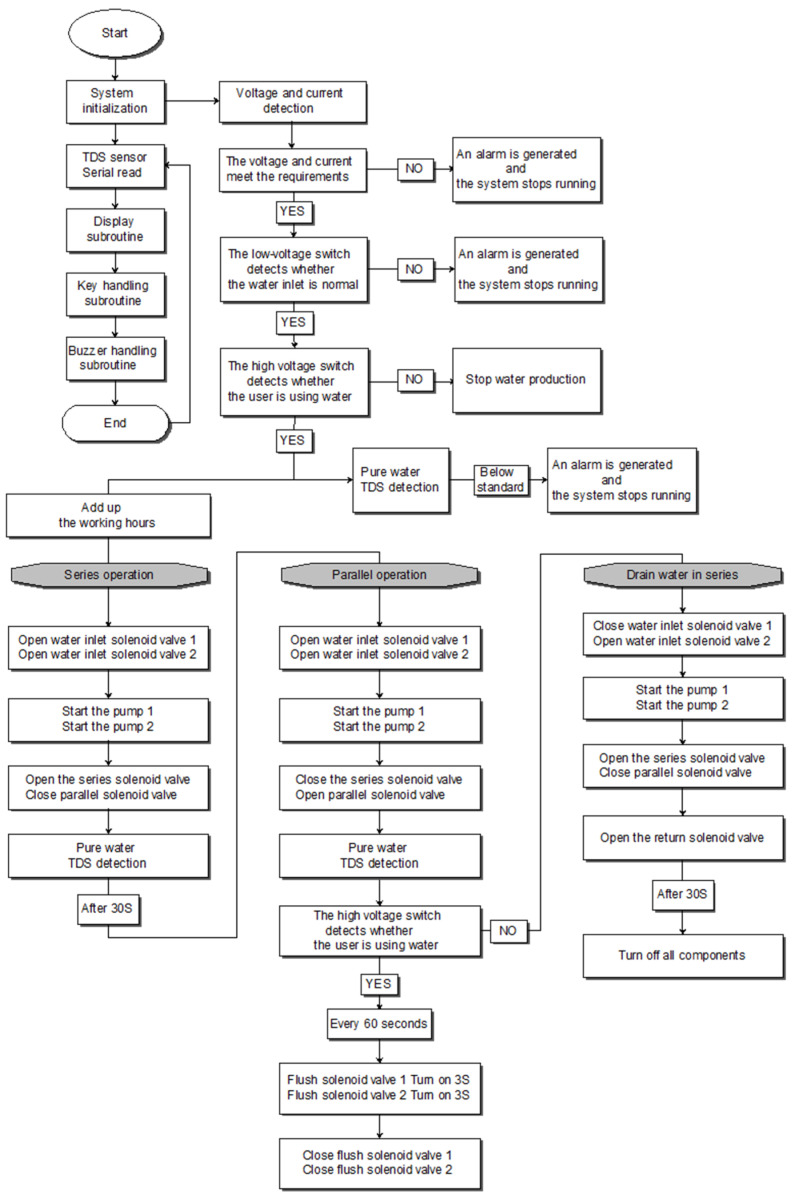
Logic Control Flowchart.

## Data Availability

The data presented in this study are available on request.
